# Supplementary material for: Inter-Versus Intra-Host Sequence Diversity of pH1N1 and Associated Clinical Outcomes
Source: Microorganisms. 2020 Jan 17;8(1):133. doi: 10.3390/microorganisms8010133 (PMC7022955; doi:10.3390/microorganisms8010133)
Supplement: Supplementary file 1 [file microorganisms-08-00133-s001.zip › Supplementary.docx]

**­­­­­­­­­Supplementary Tables and Figures**

**Supplementary Tables**

**S1 Table: Selection pressure analysis of HA gene performed using HA consensus sequences and HA haplotypes.** Analysis was done using single-likelihood ancestor counting (SLAC) method implemented in Datamonkey website (http://www.datamonkey.org). Colors of amino acids denotes their position in HA gene: Green for receptor binding site (RBS), Red for Sa antigenic site and Brown for Ca antigenic site.

| **Analyzed sequences** | **No. of sequences analyzed** | **HA subunit** | **Positively selected amino acids** |
| --- | --- | --- | --- |
| HA Consensus sequences | 90 | HA1 | 200- 201- 203 |
|  |  | HA2 | 360- 361-458 |
| HA Haplotypes | 440 | HA1 | 46-71-76-111-114-118-141-157-173-177-229-281-306 |
|  |  | HA2 | 334-340-373-380-433-437-457-467-495 |

| **Reference** | **Accession number** | **Reference** | **Accession number** |
| --- | --- | --- | --- |
| A/California/07/2009 | EPI-ISL-227813 | A/South Africa/3626/2013 | EPI-ISL-498431 |
| A/Michigan/45/2015 | EPI-ISL-227814 | A/Massachusetts/10/2013 | EPI-ISL-464861 |
| A/Slovenia/2903/2015 | EPI-ISL-237558 | A/St.Peterburg/100/2011 | EPI-ISL-320141 |
| A/Hong Kong/5659/2012 | EPI-ISL-382424 | A/Israel/Q_504/2015 | EPI-ISL-697729 |

**S2 Table: GISAID accession numbers of vaccine and representative reference viruses used in this study.**

**Supplementary Figures**

**S1 Figure:** Phylogenetic trees representing HA and NA genes of 90 pH1N1 viruses collected during 2015-2017. Clade representative viruses are indicated in red and vaccine viruses (A/California/7/ 2009 and A/Michigan/45/2015) are indicated in green. Each cluster is highlighted with respect to virus isolation year: green for 2015, blue for 2016 and gray for 2017. Scale bar indicates number of nucleotide substitutions per site per year. Phylogenetic tree was constructed using HKY+G method implemented in BEAST v1.8.4 and visualized using FigTree v1.4.

**S2 Figure:** Average percentages of synonymous and non-synonymous mutations observed among the eight genes of pH1N1 viruses sequenced between 2015 and 2017.

**S3 Figure:** Distribution, frequency and prevalence of LFVs reported in HA1 subunit **(a)** and HA2 subunit **(b)** over three-years period (2015-2017). Only validated variants shared between two or more patients are presented here. The x-axis represents amino acid position and the y-axis represents the frequencies of variants. For visual purposes, variants were scaled up, 2% to 10% was scaled up to 10%, 11% to 20% was scaled up to 20% and so on. The size of bubble indicates the prevalence of each variant among patients of each specific year. Rectangles signifies the most prevalent variants in HA head (blue) and stem (green) subunits. Most prevalent LFVs are indicated in blue and red rectangles in **a** and **b** respectively.

**S4 Figure:** Effect of genomic position, temporal distribution and prevalence of LFVs and its emergence at the consensus sequence level of HA and NA genes. No significant correlation was found between genomic position (head vs stem), temporal distribution (one-season vs two seasons), the prevalence of LFV and its appearance in consensus sequence of HA gene for both HA and NA genes. Statistical test was performed using Fisher exact test in GrapPad7.

**S5 Figure:** Localization of most prevalent LFVs in HA gene. LFVs were mainly observed in Sb antigenic site in head domain (cyan) and in the stem domain (red). Variants under positive selection are indicated with dots. Three-dimensional structure of HA monomer was downloaded from the Protein Data Bank (PDB: 3LZG) and visualized using CLC genomic workbench.

**S6 Figure:** Comparison of total number of HA haplotypes exhibited by pH1N1 positive patients. Patients were divided into three age groups: children under the age of 5, between 5 and 60 years old, and elders over the age of 60. Fisher exact test implemented in Prism7 was used to perform statistical analysis.

**S7 Figure:** Phylogenetic clustering of HA haplotypes assembled from three samples collected in 2017. Only patient #30611 -indicated in red- was suffering from respiratory complications. All HA haplotypes assembled from 30611 sample were also found in 3610 sample.

**S8 Figure:** Heat map showing polymorphic regions in HA gene of consensus and sub-consensus sequences of pH1N1 viruses (2015-2017). Polymorphic sites were identified following alignment of HA amino acid sequences extracted from quasispecies analysis (n=330 sequence). HA haplotypes were reconstructed for all HA sequences (18 nt-1680 nt) with at least 1000x coverage, and only best quality sequences with no gaps were included. Amino acid located in RBS are denoted by the gray square in middle of HA1 subunit.

**S9 Figure:** Positively selected sites in HA consensus sequences are among most prevalent LFVs. Fisher’s exact test was performed to evaluate the prevalence of LFVs in positively selected sites of all HA consensus sequences.

**S10 Figure:** Overall representation of severity score (red), number of HA haplotypes (blue) and the total number of LFVs identified in each gene of the 90 patients included in this study.
